# The clinical relevance of anti-glutamic acid decarboxylase antibodies in children with encephalitis/encephalopathy

**DOI:** 10.3389/fnins.2022.1081580

**Published:** 2023-02-02

**Authors:** Ju-Yin Hou, Hsin-Uei Liu, Cheng-Yen Kuo, Yi-Hsuan Liu, Jainn-Jim Lin, Meng-Ying Hsieh, Po-Cheng Hung, Yi-Ting Cheng, I-Chen Su, Huei-Shyong Wang, I-Jun Chou, Kuang-Lin Lin

**Affiliations:** ^1^Division of Pediatric Neurology, Department of Pediatric, Chang Gung Children’s Hospital and Chang Gung Memorial Hospital, Taoyuan City, Taiwan; ^2^College of Medicine, Chang Gung University, Taoyuan City, Taiwan; ^3^Division of Pediatric Critical Care and Pediatric Neurocritical Care Center, Chang Gung Children’s Hospital and Chang Gung Memorial Hospital, Taoyuan City, Taiwan; ^4^Chang Gung Children’s Hospital Study Group for Children with Encephalitis/Encephalopathy Related Status Epilepticus and Epilepsy (CHEESE), Taoyuan City, Taiwan

**Keywords:** encephalitis, encephalopathy, anti-glutamic acid decarboxylase antibody, GAD, immunotherapy, antineuronal antibodies, ataxia

## Abstract

Anti-glutamic acid decarboxylase (anti-GAD) antibodies are associated with different types of syndromes. However, few studies have investigated the correlation between anti-GAD antibody titers with clinical severity and outcomes in children with encephalitis/encephalopathy. In this single-center retrospective cohort study, we consecutively enrolled hospitalized children who had encephalitis and/or encephalopathy with positive anti-GAD antibodies in serum and/or cerebrospinal fluid (CSF) from February 2010 to October 2021. Thirty-seven patients were included and divided into high-titer and low-titer groups. The patients with high anti-GAD antibody titers were associated with initial symptoms of language difficulty and ataxia. The level of titers was not associated with severity or outcomes. Anti-GAD antibody titers decreased after immunotherapy, however, the clinical response to immunotherapy was variable. A transient elevation in anti-GAD antibody titers during immunotherapy was noted. Further studies are warranted to investigate the role of anti-GAD antibodies in the pathogenesis and immune mechanisms of encephalitis/encephalopathy.

## 1. Introduction

Antibodies against glutamic acid decarboxylase (GAD), the rate-limiting enzyme in the synthesis of the inhibitory neurotransmitter g-aminobutyric acid (GABA), are associated with various neurological disorders, including stiff-person syndrome, cerebellar ataxia, epilepsy, and limbic encephalitis. The pathogenic role of anti-glutamic acid decarboxylase (anti-GAD) antibodies in neurologic disorders is still unclear and currently under investigation (Graus et al., 2020).

Anti-glutamic acid decarboxylase antibody titers have been reported to be significantly higher in children with encephalitis and status epilepticus than in those of control children with therapy-resistant epilepsy (Lin et al., 2012). Presumed infectious etiologies may account for pediatric encephalitis with anti-GAD antibodies (Lin et al., 2014), suggesting a possible immune-mediated mechanism after a severe febrile infection. A previous study showed that high anti-GAD antibody concentrations were associated with specific clinical phenotypes, such as stiff-person syndrome, cerebellar ataxia, epilepsy, and limbic encephalitis (Munoz-Lopetegi et al., 2020). However, few studies have investigated the association between anti-GAD antibody titers and clinical presentation in children with encephalitis/encephalopathy.

The aim of this study was to evaluate whether serum anti-GAD antibody titers were associated with the initial symptoms, clinical severity, and outcomes of children with encephalitis/encephalopathy.

## 2. Materials and methods

### 2.1. Subjects

In this single-center retrospective cohort study, we consecutively enrolled hospitalized children who had encephalitis and/or encephalopathy with positive anti-GAD antibodies in serum and/or cerebrospinal fluid (CSF) from February 2010 to October 2021. Patients in acute, subacute, and chronic stages were all enrolled. Patients with anti-NMDA-receptor encephalitis or infectious pathogens detected in CSF were excluded. Encephalopathy was defined as the presence of at least one sign of parenchymatous brain dysfunction such as altered consciousness, personality or behavior change, seizure, paresis, or ataxia. Encephalitis was defined as the presence of encephalopathy plus at least two of the following four findings: (1) fever (body temperature > 38°C); (2) abnormal CSF examination (pleocytosis > 5 white blood cells/mL and/or increased protein content > 40 mg/dL) with negative CSF culture; (3) abnormal electroencephalogram (EEG) findings compatible with encephalitis, such as diffuse or focal slow activity, or periodic lateralized epileptiform discharges; and (4) abnormal results of neuroimaging, including computed tomography (CT) and magnetic resonance imaging (MRI) (Lin et al., 2014).

Information was collected by chart review regarding age at onset, sex, clinical symptoms (including language difficulty, ataxia, psychiatric symptoms, seizure, and/or status epilepticus through clinical observation by medical staff and caregivers), CSF analysis, EEG, neuroimaging, treatment, and clinical outcomes. CSF analysis was not compulsory. All obtained CSF samples were routinely tested for bacterial and viral cultures. Other pathogen surveys were performed in the search for etiologies, including throat swabs, rectal swabs, and serologic studies for herpes simplex virus, Epstein-Barr virus, influenza A and B, and CSF polymerase chain reaction for herpes simplex virus.

Initial EEG were categorized as negative findings, focal/diffuse cortical dysfunction, or a focal, multifocal, or generalized epileptiform discharge. Cortical dysfunction was defined as diffuse or focal theta or delta range slow waves according to their ages. The diffuse slowing can be high or low amplitude. The focal slowing can be continuous or intermittent.

Disease severity was assessed using the Glasgow Coma Scale (GCS), length of intensive care unit (ICU) stay, and Clinical Assessment Scale in Autoimmune Encephalitis (CASE) score (Lim et al., 2019). Patient outcomes were evaluated according to the modified Rankin Scale (mRS) at the most recent clinic visit. We defined mRS ≤ 2 as good functional status. Immunotherapy included intravenous methylprednisolone (IVMP) pulse therapy, intravenous immunoglobulin (IVIG), and rituximab. Immunotherapy responses were retrospectively assessed from medical files. A one-point improvement in the mRS score was considered to indicate a clinical response. Response to immunotherapy was classified as no response, partial response, and near-complete or complete response (i.e., minimal or no residual clinical signs/symptoms).

This study was approved by the Chang Gung Memorial Hospital Institutional Review Board (201104364A3, 201800500A3, 202001670A3).

### 2.2. Serum level of anti-GAD antibodies and other autoantibodies

Serum anti-GAD antibodies were tested using an enzyme-linked immunosorbent assay kit (ELISA) (RSR Ltd., Cardiff, Wales, United Kingdom). Antibody titers > 5 U/mL were considered positive. Antibody titers > 50 U/mL were enrolled as a previous study has shown anti-GAD antibody titers > 50 U/mL were evident in children with encephalitis and status epilepticus rather than therapy-resistant epilepsy (Lin et al., 2012). A radioimmunoassay (RIA) (RSR Ltd., Cardiff, Wales, United Kingdom) and cell-based assay (CBA) (Euroimmun, Lübeck, Germany) were also performed when available. CSF anti-GAD antibodies were tested by ELISA and CBA when available.

All of the patients were examined for other antineuronal antibodies, including antibodies against intercellular antigens or onconeural antigens (amphiphysin, Ma2, Ri, Yo, and Hu) (Euroimmun, Lubeck, Germany), and against neuronal surface antigens, including N-methyl-D-aspartate (NMDA) receptor, a-amino-3-hydroxy-5-methyl-4-isoxazolepropionic acid (AMPA) receptor, leucine-rich glioma inactivated 1 (LGI1) protein, contactin-associated protein 2 (CASPR2), c-aminobutyric acid-B (GABAB) receptor (Euroimmun, Lubeck, Germany), and voltage-gated potassium channel complex (VGKC) antibodies (RSR Ltd., UK). Sixteen patients were examined for anti-thyroid peroxidase (anti-TPO) antibodies. All patients underwent chest X-rays and abdominal ultrasound to assess the presence of tumors, and thyroid function were also evaluated during hospitalization and/or follow-up.

### 2.3. Statistical analysis

Fisher’s exact test was used to compare categorical variables between the high-concentration and low-concentration groups. The Mann-Whitney *U* test was used for skewed continuous variables and ordinal variables. Spearman rank-order correlation coefficient was used for correlation between ELISA and RIA. A 2-sided *p*-value less than 0.05 was considered statistically significant. All statistical analyses were performed using SPSS version 28.0 (IBM, Armonk, NY, USA).

## 3. Results

### 3.1. Laboratory tests

Among 475 patients tested for sera anti-GAD antibody, 39 children with encephalitis/encephalopathy were identified and had serum anti-GAD antibodies > 50 U/mL *via* ELISA. Two were excluded due to the diagnosis of anti-NMDA receptor encephalitis. The durations between the first measurement of anti-GAD antibodies and the onset of neurological symptoms ranged from 0 to 1,098 days, with a median of 6 days and interquartile range (IQR) 2–11 days. Anti-GAD antibody titers ranged from 51.6 U/mL to >2,000 U/mL (median 90.9 U/mL, IQR 65.1–141.5 U/mL; normal range < 5 U/mL). Only eight CSF samples were tested for anti-GAD antibodies. One was positive, and the titer was >2000 U/mL.

The sera were tested by RIA in 25 patients, and the results ranged from 0.11 to 281 U/mL (median 2.1 U/mL, IQR 1.05–2.75 U/mL; normal range < 1 U/mL). Thirty-one patients were tested by CBA. Only one was positive, and the ELISA titer was >2000 U/mL. The Spearman rank-order correlation coefficient (*r^s^*) between ELISA and RIA was 0.0264 (*p* = 0.92).

### 3.2. Clinical profiles

**FIGURE 1:**
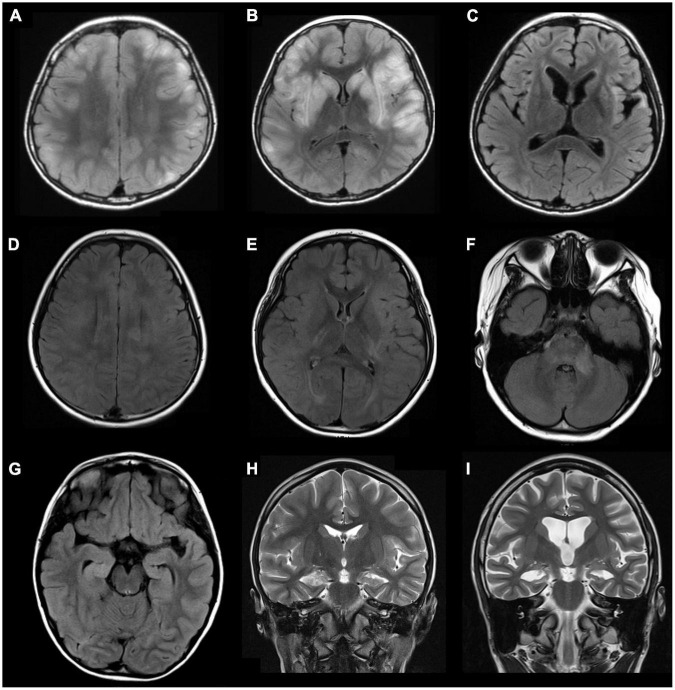
Examples of abnormal brain magnetic resonance imaging (MRI) findings in three patients with pediatric encephalitis/encephalopathy with anti-GAD antibody. Patient 1: A 10-year-old boy with clinical diagnosis of encephalitis with preceding influenza B infection 6 days ago. **(A,B)** Axial fluid-attenuated inversion recovery (FLAIR) sequences show multiple increased signals in the basal ganglia and cortical/subcortical regions of bilateral cerebral hemispheres (frontal, parietal, temporal lobes). **(C)** MRI follow-up at 9 months show progressive atrophy with stationary hyperintensities in bilateral putamen, bilateral insular regions, and left temporal tip. Patient 2: A 9-year-old girl with clinical diagnosis of rhombencephalitis with preceding HSV-1 infection 6 days ago. **(D–F)** Axial FLAIR sequences demonstrate multiple increased signals in the corpus callosum, bilateral insular regions, bilateral posterior capsules, and multiple cortical areas and white matter of bilateral hemispheres (all lobes), the brainstem, and left middle cerebellar peduncles. Patient 3: A 6-year-old girl with clinical diagnosis of febrile infection-related epilepsy syndrome (FIRES) without detected pathogen. **(G)** An axial FLAIR sequence and **(H)** a coronal T2 sequence show increased signals in right hippocampus and mesial temporal lobe. **(I)** Progressive cortical atrophy and right hippocampal atrophy were noted at the 30-month follow-up. GAD, glutamic acid decarboxylase.

Among the 37 enrolled children, 21 (56.8%) were female, with a median age of 6.5 years (IQR 3–10.5 years, range 6 months to 15 years). Seizure was the most common symptom, occurring in 30 (81.1%) of the patients. EEG was performed in 34 patients, of whom 30 (88.2%) had abnormal findings, including 24 (70.6%) with cortical dysfunction, and 17 (50%) with epileptiform discharge. CSF analysis was performed in 22 patients, of whom 12 (64.5%) had abnormal findings, including pleocytosis in 3 (13.6%), elevated CSF protein in 7 (31.8%), elevated IgG index in 2 (9.1%), and positive oligoclonal band in 1 (4.5%). Twenty-two (64.7%) of 34 patients had abnormal MRI findings, including parenchymal T2 hyperintensities over cortical/subcortical regions, white matter, hippocampal involvement, abnormal enhancement, cerebellar atrophy, and cerebral atrophy (Figure 1).

Two patients (5.4%) were diagnosed with acute disseminated encephalomyelitis (ADEM), and four patients (10.8%) were diagnosed with acute necrotizing encephalopathy of childhood (ANEC). Twenty-four (64.9%) patients were positive for serologic tests, rapid antigen detection tests, PCR, or viral cultures. The related pathogens included Mycoplasma pneumoniae in 8, influenza virus in 8 (4 with influenza A and 4 with influenza B), enterovirus in 2, herpetic simplex virus in 2, norovirus in 2, rotavirus in 1, and Salmonella enterica serogroup D in 1.

The median duration of hospitalization was 18 days (IQR 13.25–33 days, range 4–125 days). Thirty-four patients were admitted to the ICU, and the median duration of stay was 8 days (IQR 5–11.75 days, range 2-120 days).

### 3.3. Treatment, clinical severity, and outcomes

Thirty-four (91.9%) of the children received immunotherapy, and three (8.1%) did not. Among those who received immunotherapy, most received combined IVMP pulse therapy and IVIG (24, 70.5%), 2 (5.9%) received IVMP pulse therapy only, 7 (20.6%) received IVIG only, and 1 (2.9%) received combined IVMP pulse therapy, IVIG, and rituximab. None of the patients received plasma exchange.

Twenty-three (62.2%) patients had an initial GCS score ≤ 8, and the median CASE score was 12, (IQR 4–23, range 2–25). At the end of the study, 3 (8.1%) patients had died, and 9 (24.3%) had neurologic sequelae or intractable epilepsy. Twenty-eight (75.7%) patients had good functional status (mRS ≤ 2) and 9 (24.3%) had poor functional status (mRS ≥ 3).

### 3.4. Comparison between the high-titer group and low-titer group

Seventeen patients were allocated to the high-titer group (serum concentration ≥ 100 U/mL) and 20 patients to the low-titer group. The demographic data and the clinical characteristics are shown in Table 1. There were no significant differences in sex or age between the high- and low-titer groups. The high-titer group presented with more initial symptoms of language difficulty and ataxia (*p* = 0.014 and 0.033, respectively). There were no significant differences in CSF findings, EEG abnormalities, MRI abnormalities, treatment, severity, or outcomes between the two groups (Table 2). The durations between the first measurement of anti-GAD antibodies and the onset of neurological symptoms were longer in the high-titer than the low-titer group (high-titer: median 10 days, IQR 1–15 days, range 1–1098 days; low-titer: median 2 days, IQR 1–7 days, range 0–637 days; *p*-value = 0.011). After excluding four patients with duration of >15 days, the high-titer group still presented with more initial symptoms of language difficulty and ataxia (*p* = 0.026 and 0.062, respectively).

**TABLE 1:** Demographic data and clinical characteristics of the children with encephalitis/encephalopathy in the high- and low-titer anti-GAD antibody groups.

	Anti-GAD Ab < 100 U/mL (*n* = 20)	Anti-GAD Ab ≥ 100 U/mL (*n* = 17)	*P*-value
Sex			1.000
Male (%)	9 (45%)	7 (41.2%)	
Female (%)	11 (55%)	10 (58.8%)	
Age at onset (years), median (IQR)	6.3 (4.5–9.8)	7.1 (2.9–11.9)	1.000
Possible pathogen^b^	14 (70%)	10 (58.8%)	0.512
Mycoplasma	4 (20%)	4 (23.5%)	
Influenza A, B	6 (30%)	2 (11.8%)	
Herpetic simplex virus	1 (5%)	1 (5.9%)	
Other pathogens^c^	3 (15%)	3 (17.6%)	
**Initial symptoms**			
Language difficulty	1 (5%)	7 (41.2%)	0.014^a^
Ataxia	1 (5.3%)	6 (35.3%)	0.033^a^
Psychiatric symptoms	2 (10%)	5 (29.4%)	0.212
Seizure	18 (90%)	12 (70.6%)	0.212
Status epilepticus	7 (35%)	4 (23.5%)	0.495
CSF abnormal findings	8/14 (57.1%)	4/8 (50%)	1.0
CSF pleocytosis	1/14	2/8	
Elevated CSF protein	5/14	2/8	
Elevated CSF IgG index	0/4	2/3	
CSF oligoclonal band	1/4	0/2	
EEG abnormalities	17/18 (94.4%)	13/16 (81.3%)	0.326
Cortical dysfunction	16/18 (88.9%)	8/16 (50%)	0.023
Epileptiform discharge	9/18 (50%)	8/16 (50%)	1.0
MRI abnormalities	12/19 (63.2%)	10/15 (66.7%)	1.0
Other autoantibodies^d^	3	3	1.0
Hospitalization days, median (IQR)	21 (13–29)	175 (13.75–31)	0.402

Anti-GAD Ab, Anti-glutamic acid decarboxylase antibody; CSF, cerebrospinal fluid; EEG, electroencephalogram; MRI, magnetic resonance imaging; IQR, interquartile range.

^a^*p* < 0.05.

^b^Negative for cerebrospinal fluid, but detected in serum, throat, rectal, or stool sampling.

^c^Other pathogens included 1 enterovirus in both groups, 1 norovirus in both groups, 1 rotavirus in the high-titer group, and 1 Salmonella enterica serogroup D in the low-titer group.

^d^Three patients in the low-titer group had voltage-gated potassium channel complex [VGKC] antibodies. In the high-titer group, 1 patient had anti-VGKC antibodies, 1 had anti-Yo antibodies, and 1 had anti-amphiphysins antibodies.

**TABLE 2:** Treatment, clinical severity, and outcomes of the patients with encephalitis/encephalopathy of the high- and low-titer anti-GAD antibody groups.

	Anti-GAD Ab < 100 U/mL (*n* = 20)	Anti-GAD Ab ≥ 100 U/mL (*n* = 17)	*P*-value
**Clinical severity**			
CASE score, median (IQR)	19 (5.5–23)	9 (4–23)	0.842
Initial GCS, median (IQR)	7 (6–9.5)	8 (6–12)	0.333
≤8	14 (70%)	9 (52.9%)	0.328
>8	6 (30%)	8 (47.1%)	
ICU stay	19 (95%)	15 (88.2%)	0.584
Duration of ICU stay in days, median (IQR)	9 (5–13.5)	8 (5.5–9.5)	0.383
Endotracheal tube	13 (65%)	6 (35.3%)	0.103
Types of initial ASMs	2.0 ± 1.7	1.3 ± 1.4	0.151
**Treatment**			
Immunotherapy use	18 (90%)	16 (94.1%)	1.000
IVMP pulse therapy only	1 (5%)	1 (5.9%)	
IVIG only	1 (5%)	6 (35.3%)	
Combined IVMP and IVIG	15 (75%)	9 (52.9%)	
Combined IVMP, IVIG, and rituximab	1 (5%)	0	
**Outcome**			
mRS at follow-up			0.462
Good functional status (mRS ≤ 2)	14 (70%)	14 (82.4%)	
Poor functional status (mRS ≥ 3)	6 (30%)	3 (17.6%)	

Anti-GAD Ab, anti-glutamic acid decarboxylase antibody; CASE, Clinical Assessment Scale in Autoimmune Encephalitis; GCS, Glasgow Coma Scale; ICU, intensive care unit; ASM, anti-seizure medication; IQR, interquartile range; IVMP, intravenous methylprednisolone; IVIG, intravenous immunoglobulin; mRS, modified Rankin Scale.

### 3.5. Pre-treatment and post-treatment titers

**FIGURE 2:**
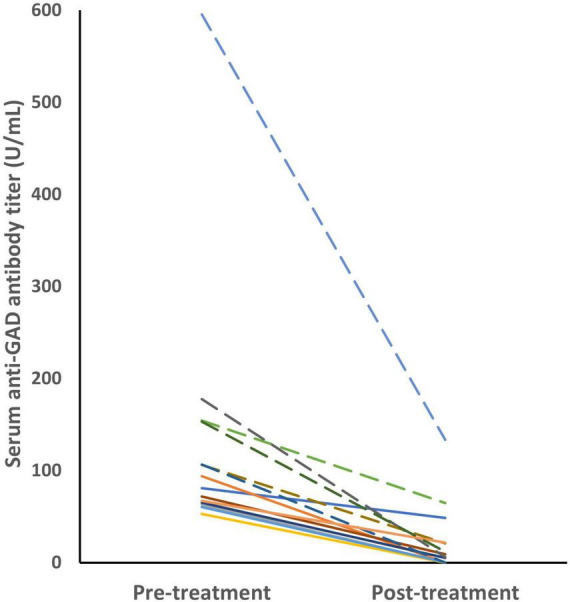
The anti-GAD antibody titers decreased after immunotherapy in 14 patients. The dashed lines represents the high-titer group. Serum anti-GAD antibodies in the high-titer group (*n* = 6) showed a median reduction of 86% (range 58–99%). The median titer reduction in the low-titer group (*n* = 8) was 95% (range 36–99%). GAD, glutamic acid decarboxylase.

Pre- and post-treatment samples were available in 14 patients (Figure 2). Serum anti-GAD antibodies in the high-titer group (*n* = 6) showed a median titer reduction of 86% (range 58–99%). The median titer reduction in the low-titer group (*n* = 8) was 95% (range 36–99%). The median change of titers were 122.8 and 60 in the high-titer and low-titer group, respectively (IQR 93.4–164.3 vs. 19.3–61.3, *p* = 0.003).

The response to immunotherapy was variable. Half of the patients (7/14) had a near-complete or complete response to immunotherapy, while 6 (43%) had a partial response with some neurologic sequelae, and 1 (7%) died.

**FIGURE 3:**
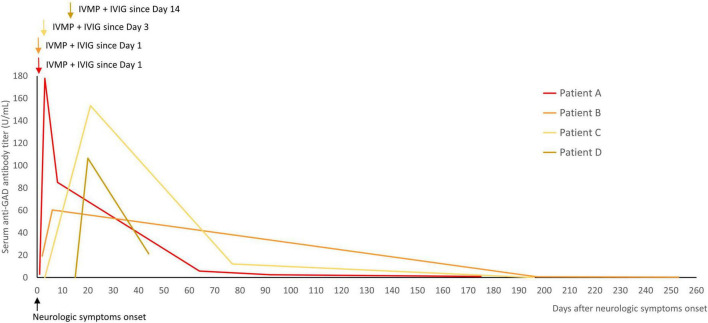
Serial changes in the titers of serum anti-GAD antibodies during immunotherapy were measured in four patients. The titers were transiently elevated during immunotherapy, and then decreased after immunotherapy during follow-up. The arrows indicated the start of immunotherapy (IVMP and IVIG in all of them). GAD, glutamic acid decarboxylase; IVMP, intravenous methylprednisolone pulse therapy; IVIG, intravenous immunoglobulin.

Four patients were tested for serial changes in serum anti-GAD antibody titers during immunotherapy (Figure 3). The results showed a transient elevation in titers during immunotherapy, and then a gradual decrease after immunotherapy during follow-up. No clinical worsening was observed during the transient elevation in titers.

### 3.6. Other autoantibodies and anti-TPO antibodies

All of the patients were tested for antineuronal antibodies. Two were positive for intracellular antigens (1 anti-amphiphysins, 1 anti-Yo) and four were positive for anti-VGKC.

Sixteen patients were tested for anti-TPO antibodies, and 11 (68.8%) were positive (median 23.1, IQR 22.0–26.2). None were diagnosed with autoimmune thyroiditis.

## 4. Discussion

In this study, we found that children with encephalitis/encephalopathy with high anti-GAD antibody titers were associated with initial presenting symptoms of language difficulty and ataxia. However, the level of the titer was not associated with severity or outcome.

Adults with high anti-GAD antibody concentrations have well-defined clinical phenotypes including stiff-person syndrome, cerebellar ataxia, epilepsy, and limbic encephalitis in adults (Baizabal-Carvallo, 2019; Munoz-Lopetegi et al., 2020), while those with low antibody concentrations present with diverse neurologic syndromes (Saiz et al., 2008; Nanri et al., 2013). In children, anti-GAD antibodies are associated with limbic encephalitis, extralimbic encephalitis including ADEM, and epilepsy (Korff et al., 2011; Mishra et al., 2014; Incecik et al., 2015; Ben Achour et al., 2018; Ren et al., 2021; Sabanathan et al., 2022). However, no previous study has compared children with encephalitis with high and low titers of anti-GAD antibodies. We found that the children with a titer > 100 U/mL had more initial symptoms of ataxia and language difficulties, although there were no significant differences in severity or outcomes. Possible explanations included the involvement of cerebellar dysfunction in the high-titer group since cerebellar ataxia was one of the most common anti-GAD antibody-related neurological disorders in adults and often present with subacute or chronic onset of gait ataxia and dysarthria with normal or mild cerebellar atrophy on behalf of the CHEESE Study Group neuroimaging (Honnorat et al., 2001). Previous pathological studies have shown selective loss of Purkinje cells with diffuse proliferation of the Bergmann glia (Ishida et al., 2007). Passive transfer experiments have demonstrated that intracerebellar administration of CSF IgGs obtained from patients with anti-GAD antibody-associated cerebellar ataxia impairs cerebellar modulation of motor control in rats (Manto et al., 2007; Mitoma et al., 2017). However, the pathogenic role of anti-GAD antibodies in cerebellar ataxia remained unclear, and the possible role of anti-GAD antibodies in cerebellar dysfunction in children with encephalitis/encephalopathy requires further studies.

Limbic encephalitis and extralimbic encephalitis including ADEM were both reported in children with anti-GAD antibodies (Incecik et al., 2015; Ren et al., 2021; Sabanathan et al., 2022). The patients in this study included those with ADEM, ANEC, and other post-infectious encephalitis. The brain MRIs in this study involve not only limbic system but also cortical/subcortical regions and white matter of extralimbic area. In addition, other autoantibodies were detected in some patients, suggesting other immune mechanisms may have contributed to the disease, and anti-GAD antibodies may present as a surrogate marker in the sera of children with different etiologies of encephalitis/encephalopathy (Budhram et al., 2021). Even though some experimental studies have suggested the pathogenic role of anti-GAD antibodies (Stagg et al., 2010; Chang et al., 2013; Hansen et al., 2013; Graus et al., 2020), this remains controversial due to the intracellular location of the GAD enzyme, and the poor correlation between the clinical manifestations and anti-GAD antibody titers, especially in stiff-person syndrome. However, our findings provide some aspects of the correlation with initial symptoms, and future studies regarding the immune mechanisms in encephalitis/encephalopathy with anti-GAD antibodies are warranted.

Previous studies have reported variable results regarding the serum concentration and treatment response. In one study, patients diagnosed with neurologic syndromes related to anti-GAD antibodies with no change in concentration after immunotherapy showed no obvious clinical improvement, while those with a reduction in antibody concentration improved clinically (Munoz-Lopetegi et al., 2020). In another two studies of stiff-person syndrome, no association was found between a reduction in titer and treatment response (Dalakas et al., 2001, 2017). In our study, all of the patients with available pre- and post-treatment samples had a reduction in antibody titer (36–99%), but with variable treatment responses. Furthermore, all four patients with available serial titer measurements during immunotherapy showed a transient elevation during immunotherapy (day 2–5) without clinical worsening. There are three possible explanations for this finding. First, the GAD enzyme was released after neuron cell death, causing more antibody production (Tsiortou et al., 2021). Second, the GAD enzyme may have transiently appeared on the cell surface in the synaptic cleft during the process of neurotransmission and exocytosis (Munoz-Lopetegi et al., 2020). Third, anti-GAD antibodies may be present in IVIG preparations (Dimitriadou et al., 2020). More frequent sampling and studies with more cases are needed to determine the timing of the highest anti-GAD antibody titer.

The limitations of this study are mainly linked to its retrospective design. First, the timing of sampling differed, and the durations between the first measurement of anti-GAD antibodies and the onset of neurological symptoms vary due to the design of the retrospective study, the limitations of lab collection, and different referral timing. Only four patients had serial titer follow-up measurements during immunotherapy. Nevertheless, our findings still provide some evidence of a transient titer elevation during immunotherapy. Second, not all sera samples were tested by RIA and CBA due to the availability of different testing methods. The weak correlation and the discrepancy between RIA and ELISA was reported in other literature comparing commercial kits of anti-GAD antibodies in the diagnosis in type 1 diabetes mellitus, probably due to the differences in assay method (Kawasaki et al., 2019; Kobra Rahmati et al., 2008; Murata et al., 2017; Oikawa et al., 2019), but both ELISA and RIA have high sensitivity for detecting GAD antibodies in type 1 diabetes mellitus. Third, the treatment differed due to a lack of consensus on treatment for encephalitis with positive anti-GAD antibodies. Although this study does not provide direct evidence for the pathogenesis of anti-GAD antibodies, further studies are warranted to investigate their potential role.

In summary, we found that anti-GAD antibody titer was associated with the initial presenting symptoms of language difficulty and ataxia, but that the level of the titer was not significantly correlated with the severity or outcome of the children with encephalitis/encephalopathy. In addition, the detection of other autoantibodies and the transient elevation in anti-GAD antibody titers during immunotherapy imply that anti-GAD antibodies may not directly trigger the pathogenesis of encephalitis/encephalopathy. Further studies are warranted to investigate the role of anti-GAD antibodies in the pathogenesis and immune mechanisms of encephalitis/encephalopathy.

## Data availability statement

The original contributions presented in this study are included in the article/supplementary material, further inquiries can be directed to the corresponding authors.

## Ethics statement

The studies involving human participants were reviewed and approved by the Chang Gung Memorial Hospital Institutional Review Board (201104364A3, 201800500A3, and 202001670A3). Written informed consent to participate in this study was provided by the participants or their legal guardian/next of kin.

## Author contributions

This research was performed at Chang Gung Children’s Hospital in Taoyuan, Taiwan. I-JC, H-SW, and K-LL conceived the study. C-YK, Y-HL, J-JL, J-YH, H-UL, Y-TC, and I-CS participated in data collection. K-LL, H-SW, and M-YH participated in study design and coordination. K-LL, H-SW, P-CH, and M-YH contributed to the patients. J-YH and H-UL drafted the manuscript. K-LL critically revised the manuscript for important intellectual content. All authors contributed to the article and approved the submitted version.
